# Post-COVID Opsoclonus Myoclonus Syndrome: A Case Report From Pakistan

**DOI:** 10.3389/fneur.2021.672524

**Published:** 2021-06-07

**Authors:** Hira Ishaq, Talha Durrani, Zainab Umar, Nemat Khan, Pamela McCombe, Mian Ayaz Ul Haq

**Affiliations:** ^1^Department of Neurology, Lady Reading Hospital, Peshawar, Pakistan; ^2^Department of Accident and Emergency, Hayatabad Medical Complex, Peshawar, Pakistan; ^3^School of Biomedical Sciences, Faculty of Medicine, The University of Queensland, Brisbane, QLD, Australia; ^4^UQ Centre for Clinical Research, Royal Brisbane and Women's Hospital, The University of Queensland, Brisbane, QLD, Australia

**Keywords:** ataxia, COVID-19, movement disorder, neurological disorder, opsoclonus myoclonus syndrome, autoimmue disease

## Abstract

**Background:** Coronavirus disease-2019 (COVID-19), caused by the severe acute respiratory distress syndrome–coronavirus-2 (SARS-CoV-2), is primarily a respiratory infection but has been recently associated with a variety of neurological symptoms. We present herewith a COVID-19 case manifesting as opsoclonus-myoclonus syndrome (OMS), a rare neurological disorder.

**Case Presentation:** A 63-year-old male diagnosed with COVID-19 infection developed behavioral changes, confusion, and insomnia followed by reduced mobility and abnormal eye movements within 48 h of recovery from respiratory symptoms associated with COVID-19. On examination, he had rapid, chaotic, involuntary saccadic, multidirectional eye movements (opsoclonus), and limb myoclonus together with truncal ataxia. CSF analysis, MRI of the brain, and screening for anti-neuronal and encephalitis related antibodies were negative. Extensive testing revealed no underlying malignancy. The patient was successfully treated with intravenous immunoglobulin (IVIG) with complete resolution of symptoms within 4 weeks of treatment.

**Conclusion:** COVID-19 infection can be associated with the manifestation of opsoclonus myoclonus syndrome, a rare neurological disorder that can be treated with IVIG if not responsive to corticosteroids.

## Practical Implications

- Following the recovery of respiratory symptoms, SARS-CoV-2 can manifest OMS, a rare neurological disorder.
- OMS manifested by SARS-CoV-2 can have a good response to IVIG in steroid-resistant cases.

## Introduction

Coronavirus disease-2019 (COVID-19), caused by the severe acute respiratory distress syndrome–coronavirus-2 (SARS-CoV-2), is primarily a respiratory infection but has been recently associated with a variety of neurological symptoms (1). Here, we present a case of post-COVID opsoclonus-myoclonus syndrome (OMS), a rare neurological syndrome characterized by a subacute onset of opsoclonus, a disorder of involuntary saccadic eye movements, ataxia, and involuntary multifocal myoclonus mainly affecting the trunk, limbs, or craniocervical region (2, 3).

## Case Description

A 63-year-old male patient, with a history of hypertension and ischemic heart disease, presented with fever, diarrhea, cough, malaise, and sore throat, with high clinical suspicion of COVID-19. The PCR for SARS-CoV-2 was positive, and the patient was managed with conservative clinical care with quarantine. The patient's COVID symptoms recovered in 3 weeks that was correlated with negative a PCR result and positive serum antibody test (IgG) for COVID-19.

Following 48 h of recovery (day 23 after the onset of COVID), the patient developed mental confusion, behavioral changes, and insomnia with a gradual reduction in mobility and abnormality in eye movements. On examination, he had rapid, involuntary saccadic multidirectional eye movements (opsoclonus) (Supplementary Video 1). Marked truncal ataxia and limb myoclonus were also present at this point (Supplementary Video 2). His baseline investigations including complete blood count, urine routine examination, thyroid profile and cerebrospinal fluid (CSF) analysis were within normal limits. Hepatitis B, C, and HIV serology were negative. Chest X-ray, ultrasound of abdomen and pelvis, and MRI of the brain were normal (Table 1). At that point, a presumed diagnosis of OMS was made. OMS is a rare disorder that is thought to be immune mediated, with primarily para-neoplastic or para-infectious etiologies. Our investigations failed to discover any malignancy or para-neoplastic condition in the patient. Additionally, the anti-neuronal antibodies profile and auto-immune encephalitis screen were also negative. Taken together, OMS manifested by the patient appeared to be associated with COVID-19 infection.

**Table 1:** Summary of patient features.

Patient's biography	•63 years •Male •Married
Initial Covid symptoms	•Fever •Diarrhea •Cough •Sore throat •Malaise
Subsequent neurological symptoms^*^	•Behavioral changes (confusion, insomnia) •Marked truncal ataxia •Myoclonus •Opsoclonus
Laboratory investigations^**^	•CBC–normal •Urine RE–normal •TFT–normal •Hep B, C, HIV Serology–negative •CSF examination–normal cell count, proteins and glucose •Anti-neuronal Anti-bodies (including Anti-CV2 Abs, Anti-Ri Abs, Anti-yo Abs, Anti-Hu Abs) profile–negative •Auto-immune encephalitis screen–negative
Neuroimaging^**^	•CT brain–normal •MRI brain–normal
Treatment and response	•^*^IV Methyl prednisolone (1 gm OD for 5 days)–no response •^**^IVIG (2 gm/Kg body weight divided over 5 days)–complete resolution of symptoms within 4 weeks

* *3 weeks after initial covid-19 symptoms*.

** All of these investigations were done after 2nd admission to hospital for neurological symptoms.

*CBC, Complete Blood Count; Urine RE, Urine Routine Examination; TFT, Thyroid Function Test; Hep B, Hepatitis B; Hep C, Hepatitis C; HIV, Human Immunodeficiency Virus; CSF, Cerebrospinal Fluid; Abs, Antibodies; CT, Computerized Tomography Scan; MRI, Magnetic Resonance Imaging; IV, Intravenous; IVIG, Intravenous Immunoglobulins*.

Before admission at our neurology ward, the patient was administered with IV methylprednisolone 1 gm OD for 5 days with no response. The patient was given IVIG treatment (2 gm/kg body weight in five divided doses) following 5 days of his last dose of methylprednisolone. Interestingly, we observed gradual but substantial improvement in OMS following IVIG treatment. The patient exhibited complete recovery over the next 4 weeks following immunotherapy (Supplementary Video 3). Subsequent follow-up of the patient to date did not show any recurrence of signs and symptoms of OMS.

## Discussion

Herein we report another case of OMS following COVID-19 infection in an adult. OMS is a rare neuroinflammatory disease of paraneoplastic, parainfectious, toxic/metabolic or idiopathic origin, characterized by opsoclonus, myoclonus, ataxia, and behavioral and sleep disorders (4). The important aspect in clinical features is that opsoclonus might not be present during the time of presentation, which can lead to delay in diagnosis or misdiagnosis as cerebellar ataxia (5).

OMS has been often reported with paraneoplastic etiology such as neuroblastoma (6, 7); however, it can manifest with parainfectious etiology, that is, following viral infections (e.g., enterovirus, Epstein–Barr virus, poliovirus, Coxsackie virus, mumps, West-Nile virus, HIV) or bacterial pathogens (e.g., Salmonella species, parasitic infections like *Plasmodium falciparum* and Mycobacterium tuberculosis) (4). Additionally, it is important to rule out any toxic-metabolic states caused by cocaine intoxication, phenytoin overdose, or hyperosmolar non-ketotic coma that may lead to OMS symptoms (4, 8). The prognosis of OMS is worse with paraneoplastic etiology, which might show resistance to immunotherapy and can lead to severe encephalopathy or death (9). In contrast, parainfectious or idiopathic OMS responds well to immunotherapy (mainly intravenous immunoglobulins or corticosteroids) (10, 11). A critical aspect in the management of OMS is to rule out underlying malignancy. In our case, there was no evidence of underlying malignancy, so suspicion of a post-infectious etiology is reasonable (4).

The emergence of neurological disorders, particularly OMS following COVID-19 infection, has been recently reported in several studies (2, 3, 12–14). Interestingly, the majority of these COVID patients developing OMS were affected by mild-to-moderate respiratory symptoms as observed in our case (2). Emamikhah et al. reported seven COVID patients affected by OMS who showed normal brain MRI scans and CSF findings as in our case (2). However, none of the patients in this case series had features of encephalopathy (2). By contrast, our patient showed behavioral changes and confusion, which has been typically reported previously with paraneoplastic OMS (4).

Our patient showed complete remission of OMS following immunotherapy within 4 weeks. Previous studies also support that OMS patients showed dramatic recovery with immunotherapy (2, 15). In agreement to our case, another study showed that a patient with generalized myoclonus and ataxia (without opsoclonus) exhibited better response to IVIG compared to high-dose methylprednisolone pulse therapy (16). However, there are reports showing recovery of OMS or myoclonus patients devoid of opsoclonus symptoms patients with IV methylprednisolone (1 g/d) (3).

The exact pathophysiology of OMS is poorly understood. It is hypothesized to have autoimmune etiology because of its response to immunosuppressive therapy, but the specific autoantibodies leading to the disorder have not yet been identified. However, it is proposed that the pathogenesis involves antibodies that react against cerebellar Purkinjie cells (17) since mild cell loss has been described in Purkinjie cell layer, inferior olives, and brainstem along with mild inflammatory changes. Hence, the diagnosis of OMS is based on clinical presentation. Furthermore, there is no specific standard or guideline for treatment, but glucocorticoids with IVIG are commonly used as first-line therapy (10).

Our case provides further evidence that COVID-19 can trigger OMS irrespective of the severity of respiratory symptoms in such patients (2, 3, 12–14). Hence, further research is needed to dissect the underlying pathophysiological mechanisms associated with COVID-19-induced neurological disorders including OMS.

## Conclusion

SARS-CoV-2 infection can lead to the manifestation of various neurological events, including a rare autoimmune OMS. This parainfectious OMS presumably caused by COVID-19 showed a dramatic response to IVIG treatment compared to corticosteroids.

## Data Availability Statement

The original contributions presented in the study are included in the article/Supplementary Material, further inquiries can be directed to the corresponding author/s.

## Ethics Statement

Ethical review and approval was not required for the study on human participants in accordance with the local legislation and institutional requirements. The patients/participants provided their written informed consent to participate in this study. Written informed consent was obtained from the individual(s) for the publication of any potentially identifiable images or data included in this article.

## Author Contributions

HI, TD, ZU, and MU identified the patient and wrote the first draft of the paper. NK and PM contributed to revising the paper. All authors revised and commented on the final draft.

## Conflict of Interest

The authors declare that the research was conducted in the absence of any commercial or financial relationships that could be construed as a potential conflict of interest.
